# Humidifier Disinfectants Are a Cause of Lung Injury among Adults in South Korea: A Community-Based Case-Control Study

**DOI:** 10.1371/journal.pone.0151849

**Published:** 2016-03-18

**Authors:** Ji-Hyuk Park, Hwa Jung Kim, Geun-Yong Kwon, Jin Gwack, Young-Joon Park, Seung-Ki Youn, Jun-Wook Kwon, Byung-Guk Yang, Moo-Song Lee, Miran Jung, Hanyi Lee, Byung-Yool Jun, Hyun-Sul Lim

**Affiliations:** 1 Department of Preventive Medicine, Dongguk University College of Medicine, Gyeongju-si, Gyeongsangbuk-do, South Korea; 2 Division of Epidemic Intelligence Service, Korea Centers for Disease Control and Prevention, Cheongju-si, Chungcheongbuk-do, South Korea; 3 Department of Clinical Epidemiology and Biostatistics, Asan Medical Center, University of Ulsan College of Medicine, Seoul, South Korea; 4 Center for Infectious Disease Control, Korea Centers for Disease Control and Prevention, Cheongju-si, Chungcheongbuk-do, South Korea; 5 Bureau of Public Health Policy, Ministry of Health and Welfare, Sejong, South Korea; 6 Department of Preventive Medicine, Asan Medical Center, University of Ulsan College of Medicine, Seoul, South Korea; 7 Department of Nursing, Asan Medical Center, University of Ulsan College of Medicine, Seoul, South Korea; 8 Department of Nursing, Hanyang University, Seoul, South Korea; 9 Korea Centers for Disease Control and Prevention, Cheongju-si, Chungcheongbuk-do, South Korea; Kaohsiung Chang Gung Memorial Hospital, TAIWAN

## Abstract

**Backgrounds:**

An outbreak of lung injury among South Korean adults was examined in a hospital-based case-control study, and the suspected cause was exposure to humidifier disinfectant (HD). However, a case-control study with community-dwelling controls was needed to validate the previous study’s findings, and to confirm the exposure-response relationship between HD and lung injury.

**Methods:**

Each case of lung injury was matched with four community-dwelling controls, according to age (±3 years), sex, residence, and history of childbirth since 2006 (for women). Environmental risk factors, which included type and use of humidifier and HD, were investigated using a structured questionnaire during August 2011. The exposure to HD was calculated for both cases and controls, and the corresponding risks of lung injury were compared.

**Results:**

Among 28 eligible cases, 16 patients agreed to participate, and 60 matched controls were considered eligible for this study. The cases were more likely to have been exposed to HD (odds ratio: 116.1, 95% confidence interval: 6.5–2,063.7). All cases were exposed to HDs containing polyhexamethyleneguanidine phosphate, and the risk of lung injury increased with the cumulative exposure, duration of exposure, and exposure per day.

**Conclusions:**

This study revealed a statistically significant exposure-response relationship between HD and lung injury. Therefore, continuous monitoring and stricter evaluation of environmental chemicals’ safety should be conducted.

## Introduction

In April 2011, a tertiary university hospital (hospital A) in Seoul reported an outbreak of adult patients with unexplained severe respiratory distress to the Korea Centers for Disease Control and Prevention (KCDC). Eight patients were women who were pregnant or in the puerperium period, and these patients had lived in different areas throughout Korea. The patients all progressed to severe pneumonia without fever, which was analogous to acute interstitial pneumonia or idiopathic acute respiratory distress syndrome.

Unfortunately, the patients did not respond to therapy, which included antiviral agents and immunosuppressive agents. Extensive microbiologic testing by the KCDC for pneumonia-related bacteria, viruses, and fungi revealed no common pathogen [1]. The patients’ clinical characteristics were different from those of known pneumonia-related diseases, and their pneumonia was suspected to be caused by a non-infectious source.

After the outbreak was widely reported by the media, other patients who were experiencing a similar illness were transferred to hospital A. Therefore, researchers in hospital A and the KCDC conducted a hospital-based case-control study. This study revealed that the presence of mold, insecticide, a humidifier, and humidifier disinfectant (HD) were significantly associated with the cases of lung injury [2]. In this context, HD has been used extensively in South Korea to disinfect humidifier water (primarily during the winter) [3]. Therefore, HD use was assumed to be the cause of the lung injury outbreak [2].

However, the hospital-based case-control study was limited by the fact that the cases resided throughout Korea, whereas the controls primarily resided in the city where hospital A was located. In addition, the exposure-response relationship between HD and lung injury was not investigated. We performed a community-based case-control study to complement the previous study and examine the exposure-response relationship between HD and lung injury.

## Materials and Methods

### Case definition and findings

The common features of the initial patients from hospital A were thoroughly reviewed by a diverse group of specialists (pulmonary internists, radiologists, pathologists, toxicologists, and infectious disease specialists) to define cases of lung injury. Based on this group’s findings, cases of lung injury were defined in a previous report as: (1) bilateral centrilobular or diffuse ground glass opacities that were observed during high-resolution computed tomography (HRCT), (2) symptoms or signs that precluded other clinical diagnoses, and (3) age of 20–54 years. Using this definition, we retrospectively reviewed hospital A’s database of HRCT images and medical records for all patients who were 20–54 years old after 2001. Patients with chronic obstructive pulmonary diseases, cancer, or a definite diagnosis of another disease were excluded. Medical records and a physical examination were used to minimize any misclassification of the cases. In addition, all suspected cases were evaluated by a pulmonologist for confirmation [2].

### Control selection and data collection

This study was approved by the Institutional Review Board of the Korea Centers for Disease Control and Prevention (no. 2011-07CON-02-P), and written informed consent was obtained from the cases (or their families) and the controls. Each case was matched with four healthy controls who did not have cough or dyspnea, according to age (±3 years), sex, residence, and history of childbirth since 2006 (for women). During August 2011, community-based controls were recruited by public health centers that were located near the residences of the patients with lung injury. A structured questionnaire was used to collect data regarding demographic characteristics, environmental exposures within the last 5 years, and type and use of humidifier and HD. The cases or their families were interviewed by the researchers, and the controls were interviewed by the staff of the local public health centers. The researchers performed additional telephone interviews with the community controls, if necessary.

### Calculating the exposure to HDs

The HDs’ ingredients can be used to create three unique groups, which are formulated using polyhexamethyleneguanidine phosphate (PHMG phosphate), 5-chloro-2-methyl-4-isothiazolin-3-one/2-methyl-4-isothiazolin-3-one (CMIT/MIT), or oligo(2-(2-ethoxy)ethoxyethyl guanidinium chloride (PGH) [4]. All cases used several liquid HD formulations that contained the same proportion of PHMG phosphate, based on the information that was supplied by manufacturers to the KCDC. The cumulative exposure to HD (in liters) was calculated by multiplying the number of HD bottles that were used each month (bottles/month), the duration of exposure to HD (months), and the volume per bottle (L/bottle). Exposure per day was calculated by dividing the cumulative exposure (mL) by the duration of exposure to HDs (days).

### Statistical analysis

The matched variables were compared using Fisher's exact test or the Mann-Whitney U test, as appropriate. Given the relatively small number of cases, exact logistic regression was then performed to calculate the risk of lung injury. If no cases or controls were included in one cell of the contingency table, the Woolf-Haldane correction (adding 0.5 to all cells) was applied to calculate the odds ratio (OR) and 95% confidence interval (CI). All statistical analyses were performed using SAS software (version 9.2; SAS institute, Cary, NC, USA), and differences with a *p*-value of <0.05 were considered statistically significant.

## Results

### Characteristics of the cases and controls

All HRCT images from 2001 to 2011 in hospital A were searched using keywords, such as centrilobular or diffuse ground glass opacities, acute interstitial pneumonia, and hypersensitivity pneumonitis. Among the approximately 1,500 images that these searches returned, 34 patients were suspected of having lung injury. Six patients were excluded based on the clinical characteristics of their disease, which were more consistent with other diseases, such as bronchiolitis obliterans organizing pneumonia, hypersensitivity pneumonitis, and acute interstitial pneumonia [2]. Among the 28 eligible cases, 16 patients or their families agreed to participate in this study. Among the 64 matched controls, 4 patients were excluded because of their age or a history of childbirth; thus, 60 controls were included in the final analysis.

**Fig 1:**
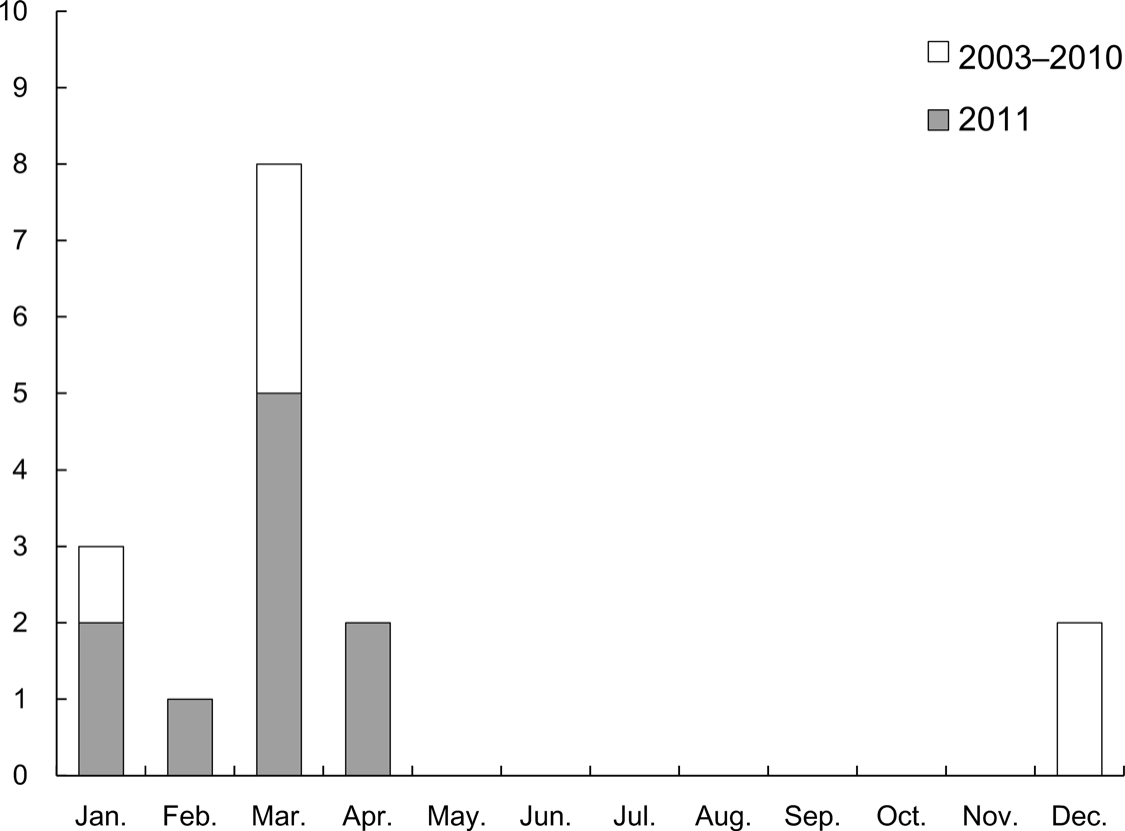
Number of cases with lung injury from one hospital in Seoul.

The cases consisted of 3 men and 13 women, and the median age of the cases was 36 years (range: 28–49 years). There were no significant differences between the characteristics of the cases and controls (Table 1). Using dyspnea to define the onset of lung injury, 10 cases (62.5%) occurred during 2011, and all of these cases occurred during the winter (37.5%) and spring (62.5%) (Fig 1). Three patients (18.8%) ultimately died, and four patients (25.0%) underwent lung transplantation. Among the 12 patients who lived with their children, the children of 4 patients (33.3%) experienced dyspnea.

**Table 1:** Socio-demographic and clinical characteristics of the study participants.

	Cases (n = 16)	Controls (n = 60)	*P-value*^a^
Age (years)	36 (28–49)	35 (27–51)	0.49
Sex			
Women	13 (81.3)	49 (81.7)	1.00
Men	3 (18.8)	11 (18.3)	
Residence			
Seoul	4 (25.0)	14 (23.3)	1.00
Other	12 (75.0)	46 (76.7)	
History of childbirth (women)^b^			
Yes	13 (100.0)	49 (100.0)	1.00
No	0 (0.0)	0 (0.0)	
Marital status			
Married	15 (93.8)	57 (95.0)	1.00
Not married	1 (6.3)	3 (5.0)	
Smoking			
Current or ex-smoker	3 (18.8)	4 (6.7)	0.16
Non-smoker	13 (81.3)	56 (93.3)	
Past medical history			
Diabetes mellitus	0 (0.0)	0 (0.0)	1.00
Hypertension	0 (0.0)	0 (0.0)	1.00
Asthma	0 (0.0)	1 (1.7)	1.00
Allergic rhinitis	2 (12.5)	12 (20.0)	0.72

Data are reported as number (%) or median (range).

^a^Fisher's exact test or the Mann-Whitney U test was used, as appropriate.

^b^Percentages are calculated as a proportion of the women in the group.

### Association of lung injury with environmental exposure

The cases were significantly more likely to have been exposed to HDs, compared to the controls (OR: 116.1, 95% CI: 6.5–2,063.7). For HDs containing PHMG phosphate, the cases were 203.8-fold (95% CI: 11.1–3,724.1) more likely to have been exposed, compared to the controls. However, humidifier use was not significantly associated with lung injury (OR: 2.9, 95% CI: 0.7–12.9). Among the house-related factors and housewares, exposures to mold (OR: 1.3, 95% CI: 0.4–4.8) and insecticide (OR: 2.9, 95% CI: 0.7–12.9) were also not significantly associated with the lung injury. Furthermore, the controls were significantly more likely to have purchased new furniture (OR: 0.1, 95% CI: 0.0–0.6) and hung new wallpaper (OR: 0.1, 95% CI: 0.0–0.4), compared to the cases (Table 2).

**Table 2:** Comparing the environmental exposures between the cases and controls.

	Cases (n = 16)		Controls (n = 60)		Odds ratio (95% CI) ^a^			
Residence-related								
Close to dangerous facilities (2 km)	4/11 (36.4)		12/58 (20.7)		2.2 (0.4–10.3)			
Close to main roads (100 m)	7/11 (63.6)		35/60 (58.3)		1.3 (0.3–6.5)			
Quality of air (bad)	2/11 (18.2)		17/60 (28.3)		0.6 (0.1–3.2)			
House-related								
Moved into a new house	5/16 (31.3)		26/60 (43.3)		0.6 (0.1–2.2)			
Repaired house^b^	0/13 (0.0)		14/60 (23.3)		0.1 (0.0–2.1)			
Purchased new furniture	3/13 (23.1)		42/60 (70.0)		0.1 (0.0–0.6)			
Hung new wallpaper	1/13 (7.7)		37/60 (61.7)		0.1 (0.0–0.4)			
Sofa	7/13 (53.9)		42/60 (70.0)		0.5 (0.1–2.1)			
Carpet	1/13 (7.7)		20/60 (33.3)		0.2 (0.0–1.3)			
Mold	10/16 (62.5)		34/60 (56.7)		1.3 (0.4–4.8)			
Appliances								
Air conditioner	7/12 (58.3)		41/60 (68.3)		0.7 (0.2–3.0)			
Air cleaner	2/13 (15.4)		18/60 (30.0)		0.4 (0.0–2.3)			
Ozone cleanser^b^	0/16 (0.0)		1/60 (1.7)		1.2 (0.1–30.9)			
Humidifier^b^	16/16 (100.0)		41/60 (68.3)		15.5 (0.9–272.0)			
Humidifier disinfectant^b^								
All types	16/16 (100.0)		13/60 (21.7)		116.1 (6.5–2,063.7)			
PHMG phosphate	16/16 (100.0)		8/60 (13.3)		203.8 (11.1–3,724.1)			
Housewares								
Air freshener	4/13 (30.8)		11/60 (18.3)		2.0 (0.4–8.8)			
Scented candle/fumigant	1/13 (7.7)		8/60 (13.3)		0.6 (0.0–4.8)			
Insecticide	8/13 (61.5)		21/60 (35.0)		2.9 (0.7–12.9)			
Hairspray	2/13 (15.4)		4/60 (6.7)		2.5 (0.2–20.2)			
Others								
Cleaned bathroom with bleach	8/11 (72.7)		28/57 (49.1)		2.7 (0.6–17.6)			
Boiled clothes with detergent	9/13 (69.2)		43/60 (71.7)		0.9 (0.2–4.5)			
Visited laundromat	12/13 (92.3)		57/60 (95.0)		0.6 (0.1–35.9)			

Data are reported as number (%).

CI, confidence interval. PHMG phosphate, polyhexamethyleneguanidine phosphate.

^a^Exact logistic regression was used.

^b^Haldane correction was used.

### HDs containing PHMG phosphate and lung injury

The cases exhibited significantly greater exposure to HDs containing PHMG phosphate, compared to the controls (*P* < 0.01). However, lung injury was not associated with exposure to HDs containing CMIT/MIT (*P* = 1.00) or PGH (*P* = 1.00). The median time from the first use of HDs containing PHMG phosphate to the onset of lung injury was 2.1 years (range: 0.1–8.4 years).

The cases stayed in a room with an operating humidifier for a median period of 8 h (range: 7–24 h), compared to a median period of 9 h for the controls, who used HDs containing PHMG phosphate (range: 1–24 h) (*P* = 0.95). Within the last 5 years, the cases used 8.2 L (range: 0.4–28.0 L) of HDs containing PHMG phosphate for 10.5 months (range: 1.0–30.0 months). In contrast, only 8 controls used 0.4 L (range: 0.1–4.7 L) of HDs containing PHMG phosphate for 6.0 months (range: 3.0–24.0 months). Based on these data, we classified the cumulative exposure to HDs containing PHMG phosphate as <0.5 L, 0.5–<2.5 L, and ≥2.5 L; the duration of exposure was classified as <5 months, 5–<10 months, and ≥10 months. The risk of lung injury increased with increasing cumulative exposure and duration of exposure, with an exposure of 0.5–<2.5 L providing an OR of 76.0 (95% CI: 2.5–∞) and an exposure of ≥2.5 L providing an OR of 272.9 (95% CI: 25.7–∞) (Table 3).

**Table 3:** Exposure-response relationship between humidifier disinfectants containing polyhexamethyleneguanidine phosphate and lung injury.

	Cases (n = 16)	Controls (n = 60)	Odds ratio (95% confidence interval)^a^
Cumulative exposure within last 5 years (L)				
<0.5	1 (1.7)	57 (98.3)	Reference
0.5–<2.5	2 (66.7)	1 (33.3)	76.0 (2.5–∞)
≥2.5	13 (86.7)	2 (13.3)	272.9 (25.7–∞)
Duration of exposure during the last 5 years (months)			
<5	4 (6.9)	54 (93.1)	Reference
5–<10	3 (42.9)	4 (57.1)	9.5 (1.0–83.0)
≥10	9 (81.8)	2 (18.2)	52.9 (7.8–664.0)
Exposure per day (mL/day)			
<10	3 (4.8)	59 (95.2)	Reference
10–<20	6 (85.7)	1 (14.3)	95.4 (8.4–>999)
≥20	7 (100.0)	0 (0.0)	133.5 (16.7–∞)

Data are reported as number (%).

^a^Exact logistic regression was used.

The cases used 16.7 mL of HDs containing PHMG phosphate per day (range: 7.3–133.3 mL/day), whereas the controls used 1.8 mL of HDs containing PHMG phosphate per day (range: 0.7–12.9 mL/day). The risk of lung injury also increased with increasing exposure per day, with an exposure of 10–<20 mL per day providing an OR of 95.4 (95% CI: 8.4–>999) and an exposure of ≥20 mL per day providing an OR of 133.5 (95% CI:16.7–∞) (Table 3).

## Discussion

In this study, cases of lung injury were associated with exposure to HD, but not with other environmental exposures (e.g., humidifier, mold, and insecticide) that were significantly associated with lung injury in the hospital-based case-control study [2]. The risk of lung injury increased with greater exposure to HDs containing PHMG phosphate. Therefore, the present community-based case-control study revealed a significant exposure-response relationship between HD and lung injury.

In South Korea, the proportions of humidifier and HD use among healthy adults during 2012 were 37.2% and 18.1%, respectively, and the proportions among <49-year-old individuals were 54.3% and 23.9%, respectively [3]. In the present study, the controls exhibited a similar age range (27–51 years), and similar proportions of humidifier and HD use (68.3% and 26.3%, respectively). In contrast, only 28.1% of American families with children use humidifiers [5], and French families with neonates rarely use humidifiers [6]. Thus, it appears that humidifier use in South Korea is higher than that in other countries. Interestingly, we could not find any previous non-Korean research regarding HDs, and, to our knowledge, HDs have only been developed and used in South Korea.

During late 2011 (after the lung injury outbreak), the Korean Institute of Toxicology performed an inhalation study of HDs containing PHMG phosphate, CMIT/MIT, or PGH. In that study, the rats that were exposed to PHMG phosphate or PGH (via whole-body inhalation) exhibited dyspnea and biopsy-confirmed pulmonary fibrosis. However, the rats that were exposed to CMIT/MIT did not exhibit dyspnea or pulmonary fibrosis [4].

The first HD in South Korea was formulated using CMIT/MIT and was introduced in 1994, and the first HD to contain PHMG phosphate was introduced before 2000. Based on manufacturer data that were provided to the KCDC, it appears that most HDs in South Korea are formulated using CMIT/MIT or PHMG phosphate. Only HDs containing PHMG phosphate were used by the patients with lung injury in the present study, although 2 eligible patients who declined to participate were exposed to HDs containing PGH. Nevertheless, it is possible that there have been other outbreaks of lung injury due to HD exposure, given the fact that these products have been available since 1994. For example, an outbreak of children’s interstitial lung disease (chILD), which is analogous to lung injury, occurred among 15 children during the spring of 2006 [7]. In addition, a hospital-based case-control study of chILD in 2012 revealed that HD exposure was associated with an increased risk of chILD [8]. Similarly, we observed that of 4 of the patients with lung injury had children who also experienced dyspnea (a symptom of lung injury).

Having newly hung wallpaper and newly purchased furniture exhibited an inverse association with lung injury. However, the exposure period of the cases (within the last 5 years) was not equal to that of the controls (2007–2011). This difference might influence these results, as the cases had a greater likelihood of having young infants during the exposure period, which might have made it difficult for them to hang new wallpaper or purchase new furniture.

On August 31, 2011, the KCDC announced that HD might be the cause of the lung injury outbreak, and recommended that HD use should be limited [9]. Nevertheless, all patients and controls had completed the questionnaires before this announcement, which precludes the possibility that the public announcement might have biased our findings. Furthermore, all sales of HDs were suspended on November 11, 2011, and several HDs containing PHMG phosphate or PGH were subsequently recalled [10]. Prospective hospital-based monitoring did not reveal any new cases of lung injury during autumn 2011 and autumn 2012, which further implicates HD as a cause of lung injury [11].

Among the 9 survivors of lung injury who did not undergo lung transplantation, 6 survivors had follow-up HRCT images from 1 year after the onset of their lung injury. The HRCT images revealed mild decreases (3 cases) or no changes (3 cases) in the extent of the centrilobular or diffuse ground glass opacities. These findings suggest that the patients’ conditions improved slightly after stopping the use of the HDs, although residual lung fibrosis was still present.

There were several limitations in this study. First, we only evaluated cases at a single hospital, and no other possible cases were included. The large ORs and broad CIs for HDs exposure might be influenced by the small sample size, and should be interpreted with caution. Second, the community controls were evaluated using a questionnaire survey that was administered by local health workers, due to limited time and resources. Nevertheless, additional telephone interviews were performed by the researchers to confirm missing or unclear responses. Third, HRCT could not be performed to exclude lung injury among the community controls, although the controls in this study only included healthy people without cough and dyspnea.

In conclusion, the present case-control study provided additional evidence that HD is a cause of lung injury. Therefore, environmental chemicals can pose unanticipated risks to human health, and continuous monitoring and more strict safety evaluations should be performed for environmental chemicals.

## Supporting Information

S1 Table Raw data regarding the exposure-response relationship between humidifier disinfectants containing polyhexamethyleneguanidine phosphate and lung injury. This is the S1 Table legend.
(XLSX)
